# Toward Personalized Medicine Approaches for Parkinson Disease Using Digital Technologies

**DOI:** 10.2196/47486

**Published:** 2023-09-27

**Authors:** Amit Khanna, Graham Jones

**Affiliations:** 1 Neuroscience Global Drug Development Novartis Pharma AG Basel Switzerland; 2 GDD Connected Health and Innovation Group Novartis Pharmaceuticals East Hanover, NJ United States; 3 Clinical and Translational Science Institute Tufts University Medical Center Boston, MA United States

**Keywords:** digital health, monitoring, personalized medicine, Parkinson disease, wearables, neurodegenerative disorder, cognitive impairment, economic burden, digital technology, symptom management, disease control, debilitating disease, intervention

## Abstract

Parkinson disease (PD) is a complex neurodegenerative disorder that afflicts over 10 million people worldwide, resulting in debilitating motor and cognitive impairment. In the United States alone (with approximately 1 million cases), the economic burden for treating and caring for persons with PD exceeds US $50 billion and myriad therapeutic approaches are under development, including both symptomatic- and disease-modifying agents. The challenges presented in addressing PD are compounded by observations that numerous, statistically distinct patient phenotypes present with a wide variety of motor and nonmotor symptomatic profiles, varying responses to current standard-of-care symptom-alleviating medications (L-DOPA and dopaminergic agonists), and different disease trajectories. The existence of these differing phenotypes highlights the opportunities in personalized approaches to symptom management and disease control. The prodromal period of PD can span across several decades, allowing the potential to leverage the unique array of composite symptoms presented to trigger early interventions. This may be especially beneficial as disease progression in PD (alongside Alzheimer disease and Huntington disease) may be influenced by biological processes such as oxidative stress, offering the potential for individual lifestyle factors to be tailored to delay disease onset. In this viewpoint, we offer potential scenarios where emerging diagnostic and monitoring strategies might be tailored to the individual patient under the tenets of P4 medicine (predict, prevent, personalize, and participate). These approaches may be especially relevant as the causative factors and biochemical pathways responsible for the observed neurodegeneration in patients with PD remain areas of fluid debate. The numerous observational patient cohorts established globally offer an excellent opportunity to test and refine approaches to detect, characterize, control, modify the course, and ultimately stop progression of this debilitating disease. Such approaches may also help development of parallel interventive strategies in other diseases such as Alzheimer disease and Huntington disease, which share common traits and etiologies with PD. In this overview, we highlight near-term opportunities to apply P4 medicine principles for patients with PD and introduce the concept of composite orthogonal patient monitoring.

## Introduction

Parkinson disease (PD) is a debilitating neurodegenerative disorder with age-related onset [1]. In the United States alone, close to 100,000 new patients are currently diagnosed with PD each year, representing a sharp increase from premillennium estimates and aligning with general trends of an aging population and increased life expectancies [2]. The incidence of PD is higher in women than in men and there appear to be regional and geographic variations, suggesting that the disease pathogenesis may be influenced by a combination of genetic, epigenetic, and environmental factors [3]. The average age at diagnosis is 60 years; however, the prodromal period can present up to 20 years before a formal diagnosis, which offers potential for interventions to delay or even prevent onset [4]. As a neurodegenerative disease, there are some similarities of PD with related disorders, including Alzheimer disease (AD) and Huntington disease (HD) (Table 1) [5].

**Table 1:** Comparative targets and assessment tools across major neurodegenerative diseases [6].

Targets and tools	Alzheimer disease	Parkinson disease	Huntington disease
Age of diagnosis (years)	60	60	40
Induction period (years)	15	20	10
Target proteins	Amyloid-β, tau	α-Synuclein (SNCA)	Huntingtin (HTT)
Genetic markers	*APOE*^a^, *PSEN*^b^	*LRRK*^c^, *PARK*^d^, *PINK*^e^, *SNCA*	*HTT*
Symptomatic treatment	None	L-DOPA^f^, istradefylline	None
Primary impairment	Memory	Movement	Movement
Rating scales	iADRS^g^, ADAS-Cog^h^	MDS-UPDRS^i^, H&Y^j^, ADL^k^, PDQ-39^l^	UHDRS^m^

^a^APOE: apolipoprotein E.

^b^PSEN: presenilin-1.

^c^LRRK: leucine-rich repeat kinase 2.

^d^PARK: parkin.

^e^PINK: PTEN-induced kinase.

^f^L-DOPA: levodopa (L-3,4-dihydroxyphenylalanine).

^g^iADRS: Integrated Alzheimer's Disease Rating Scale.

^h^ADAS-Cog: Alzheimer’s Disease Assessment Scale-Cognitive subscale.

^i^MDS-UPDRS: Movement Disorder Society-Unified Parkinson’s Disease Rating Scale.

^j^H&Y: Hoehn and Yahr scale.

^k^ADL: Activities of Daily Living.

^l^PDQ-39: Parkinson’s Disease Questionnaire.

^m^UHDRS: Unified Huntington’s Disease Rating Scale.

Genetic and molecular targets have been established for all three diseases, and advances in molecular imaging, biofluid analysis, and exploratory biomarkers (including cerebrospinal fluid [CSF] and serum neurofilament light chain, sebum-based amyloid-β_1-42_, acoustic signatures) are helping to reduce the reliance on traditional analyses, in turn is expanding our understanding of disease progression [7].

PD differs from AD and HD in that (1) there is an extended prodromal period and (2) the availability of symptomatic therapies for the treatment of motor symptoms allows the capture of potentially rich information on symptomatic management [8]. To this end, several high-profile registries have been established, enabling sharing insight in real time on progression and providing useful health outcome assessments. Principal among these are the Oxford Discovery [9], Tracking UK [10], and Parkinson's Progression Markers Initiative (PPMI) [11] cohorts, and an integrated database has been established by the Critical Path Institute to allow users seamless access to standardized data [12].

## Disease Progression and Management

**Figure 1:**
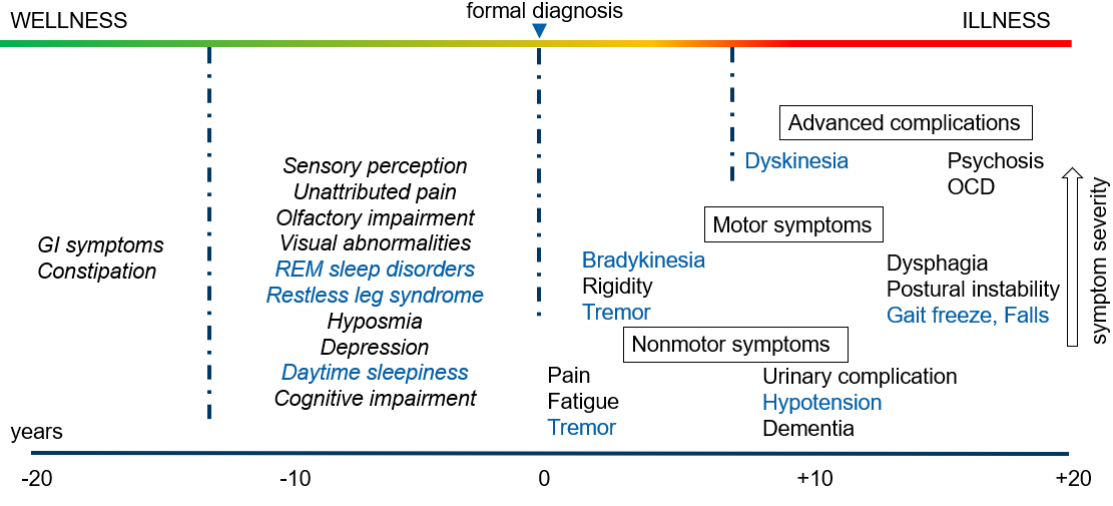
Symptomatic progression of Parkinson disease from prodromal through diagnostic stages [13]. GI: gastrointestinal; OCD: obsessive compulsive disorder.

The disease trajectory for patients with PD is complex, involving motor and nonmotor manifestations, compounded by motor and neurobehavioral complications at later stages [13] (Figure 1). Of significance, many manifestations are observed in the prodromal stages and can be useful indicators to stimulate patient awareness and subsequent interventions, including accurate diagnosis. Given the association of a number of well-defined genetic markers with PD, screening and genetic counseling may also offer opportunity for the deployment of specific monitoring approaches for at-risk individuals at a presymptomatic stage [14]. In particular, the rapid development of consumer-grade sensors and biometric devices may be useful in detecting signature events and more importantly offering a means to track them longitudinally over extended time periods [15]. For example, wrist-worn devices can monitor sleep profiles and irregular motor movements, and, if tied to a health-related app that surveys other parameters (eg, olfactory), might serve as an early alert. Commercially available smartwatches with three-axis ballistographic sensors and photoplethysmography technologies (eg, Apple iPhone) are currently able to capture signals relevant to PD (Figure 1, blue text) and the potential exists for patients who are diagnosed to switch from passive to active monitoring, allowing surveillance of other signals (eg, voice-based signals, which might detect the severity of dysphagia and fatigue) [16].

## Clinical Analysis of PD Patient Populations

**Figure 2:**
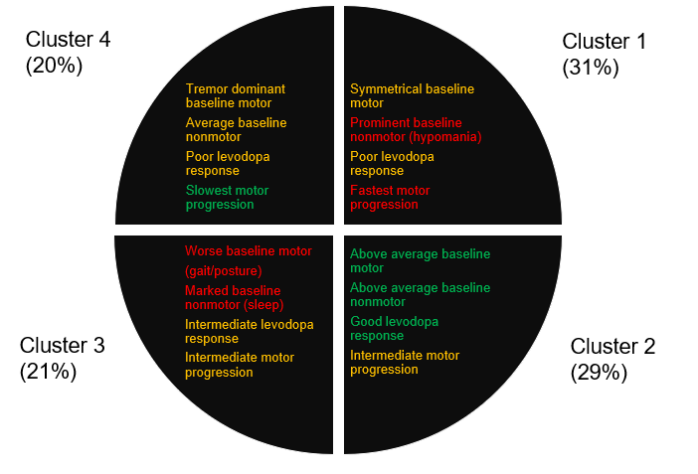
Clustering of disease progression in patients with Parkinson disease observed in the UK Tracking Parkinson’s cohort [15].

Numerous clinical studies are underway through a variety of cohorts and registries established by government, foundations, and nonprofit organizations. A number of different and distinct patient phenotypes have been proposed, which may be a consequence of the genetic contributors of the disease; crossover with comorbid neurodegenerative diseases such as AD; and lack of clear definition on etiology, including ties to Lewy body dementia (LBD). In an analysis of the 769-patient Oxford Parkinson Disease Centre discovery cohort, Lawton et al [17] proposed a total of five distinct phenotypic patient subgroups (see Table 2, where a higher score represents a worsening effect). In subsequent studies involving the UK Tracking Parkinson’s cohort, four discrete patient clusters were suggested (Figure 2), which present with widely differing motor and nonmotor impairments and progression rates along with varying degrees of response to the standard-of-care symptomatic therapy, levodopa (L-DOPA) [6]. Origins of the subgroupings were reasoned to be based on dopaminergic-resistant features that when coupled with measures of dopamine-responsive motor activities provide the basis for study of progression and early stage treatment response [18]. Based on these observed heterogeneities in clinical presentation and progressions, there are sustained efforts to track phenotyped cohorts longitudinally. A very recent study examined patients across the UK Tracking PD cohort (N=1807), the Oxford Discovery cohort (N=842), and PPMI cohort (N=472) using mixed modeling methods including genetic factors [19]. The Bayesian phenotypes developed (from longitudinal data spanning 5-10 years) were characterized in the form of three “axes,” the most influential of which (axis 1) was associated with an increased risk for AD and upregulation of microglia-expressed genes, which may imply involvement of neuroinflammatory pathways [19]. This axis presents with lowered levels of CSF amyloid-β_1-42_, severe baseline motor and nonmotor features, and with a higher likelihood of progression to early dementia. The availability of these patient cohorts is playing an invaluable role in the quest to standardize disease characterization, tracking, and management, but clearly indicate that unified approaches to assessment and treatment may not be appropriate or even desirable. There is also considerable effort being applied to evaluate the biological underpinnings of PD and challenging established hypotheses in the bid to develop effective therapeutics for the management of symptoms [20].

**Table 2:** Phenotypic subgroups of Parkinson disease and specimen features identified in the Oxford Parkinson Disease Centre cohort [17].

Group	Frequency (%)	Tremor^a^	Smell^a^	Cognition^a^
Mild motor and nonmotor disease	25.4	–2	–2	–2
Poor posture and cognition	23.3	–2	–2	+2
Severe tremor	20.8	+4	0	–1
Poor psychological well-being, RBD^b^, and sleep	18.9	–2	+3	–2
Severe motor and nonmotor disease and poor psychological well-being	11.7	+2	+5	+5

^a^A higher score represents a worsening effect.

^b^RBD: REM sleep behavior disorder.

## Drug Targets for PD

As noted in Table 1, α-synuclein remains a primary target for therapeutic drug development, either through reducing its production, propensity to aggregate, or ability to spread extracellularly. Major clinical evaluations of monoclonal antibodies targeting α-synuclein have been reported, including prasinezumab developed by Roche [21] and cinpanemab from Biogen [22]. Although these clinical trials did not meet primary endpoints, the prasinezumab trial had a promising signal relating to motor function, prompting Roche to revisit their clinical development strategy with an additional phase II trial [23]. One unresolved question relates to the precise role of α-synuclein itself. Aggregates of misfolded α-synuclein accumulate in Lewy bodies (a hallmark of the disease); yet, determining the exact mechanism underlying this effect and identification of the toxic form of the protein remain a challenge, with some theorizing that these deposits may even be protective [24]. In terms of impairment of axonal transport (a key detriment in patients with PD), at least three other proteins are known to play roles, including amyloid and tau (primary targets for AD research) and the transactive response DNA-binding protein TDP43, albeit by differing mechanisms [25]. Indirect targeting of α-synuclein aggregation may also prove fruitful. Early studies showed that Annovis’ buntanetap (which targets an iron response element in mRNAs encoding proteins found in Lewy bodies) had the effect of lowering aggregation of α-synuclein and improving cognition of patients with PD and AD [26]. A 450-person phase III trial in patients with early stage PD will read out in 2023 and may bolster interest in this approach [26]. Additional strategies include inhibiting other neuroinflammatory pathways [27], correcting impaired autophagy [28], addressing proteasome dysregulation [29], and repairing defective lysosomes [30]. One of these approaches, targeting dysfunctional mitochondria in PD [31], is being actively pursued with drug candidates developed by Lucy Therapeutics, NRG Therapeutics, and Pretzel Therapeutics, among others [32]. Based on potential overlap between PD and AD, the general interplay between neurodegenerative disease pathways is receiving considerable attention, and in this regard LBD is highly relevant [33].

Although anatomic and histopathologic traits are reminiscent of those encountered in PD, LBD presents symptoms reflecting both AD and PD. Cognitive decline is typically pronounced, often more so than movement impairment, but is often accompanied by psychotic episodes and falls, prompting debate on whether it is a dementia-dominant variant of PD or a different condition [34]. The detection of elevated levels of amyloid-β plaques in the brains of patients with LBD suggests that it is a more defined disease as patients show wide-ranging responses to antipsychotics and cognitive enhancers used in the treatment of both PD and AD [35]. Patients with PD and AD also commonly suffer from anxiety, sleep disturbance, and depressive episodes, and new symptomatic therapeutic and behavioral approaches to these ailments are needed in addition to those addressing causative factors [36]. The similarities and differences between the disease etiology of PD, AD, and LBD coupled with genetic, epigenetic, lifestyle, and environmental factors drives the need for personalized approaches to disease management.

## Preventive and Personalized Approaches to PD

There is compelling evidence that correlates improved outcomes when patients play an active, participatory role in their health management. Further research has identified specific patient phenotypes that are likely to respond more favorably due to higher levels of engagement [37]. In the case of PD, it is relevant to consider factors that benefit the patient in the extended prodromal phase and subsequently following disease onset or diagnosis. The prodromal period can exceed two decades, and given the fluid debate on causative and influencing factors, there may be actions and behaviors that could potentially delay onset, including dietary regimens, environmental location, and degree of physical activity. Likewise, following initial diagnosis, the potential to delay progression (or the rate) of the disease could be influenced by the patient. The social and economic impact of delaying onset by merely 1 year is substantial and could form the basis of coverage decisions for therapeutics made by payers [38]. For these reasons, the merits of adopting personalized medicine (PM) approaches over the patients’ health span are interesting to consider. One of the most developed frameworks for PM was articulated as “P4 medicine” by Leroy Hood and his team at the Institute of Systems Biology [39] in which the 4 Ps represent *predictive, preventive, personalized,* and *participatory* components of medicine. This approach could overlay well in PD as there are genetic associations (*predictive*) and studies that infer that delayed onset/progression might be achieved through lifestyle, dietary factors, and potentially therapeutics (*preventive*).

**Figure 3:**
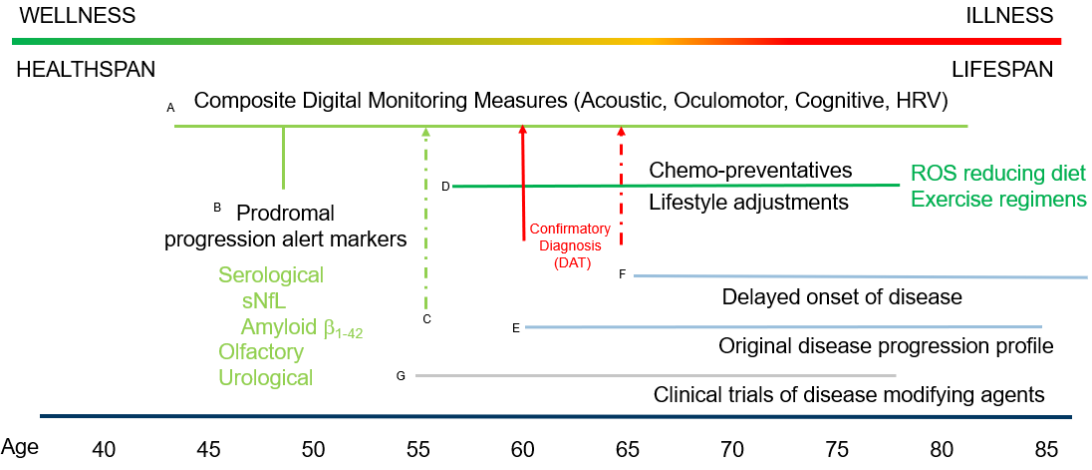
Illustrative approach to components of P4 (predict, prevent, personalize, and participate) medicine in Parkinson disease management. DAT: dopamine transporter; HRV: heart rate variability; ROS: reactive oxygen species; serum sNfL: serum neurofilament light chain.

The highly fragmented nature of the PD patient cohorts described above supports individualized approaches to patient care (*personalized*) and improved outcomes (eg, cognitive score, motor function) when patients (and in later stages of the disease with caregivers) actively engage in the treatment plans (*participatory*). The components are illustrated in Figure 3 where objectives would be to delay the onset of motor dysfunction through use of early warning signals (A, B) to prompt lifestyle factor changes (D) and earlier access to therapeutics once prodromal signals are confirmed (C). If successful, such an approach could serve to delay onset of the diseased state (E-F) and also stimulate clinical trials of new therapeutics (G). The early detection methods could be a combination of behavioral (Figure 1), serological and biologic analytes, and digital measures. Under the P4 approach, this would then trigger best actions on the patient’s part, including accessing dedicated care services, engaging in a physical activity plan (specific exercise regimens are associated with slowing PD progression), and adhering to specific dietary recommendations (eg, high-antioxidant diets to ameliorate the impact of reactive oxygen species) [40]. This period would then represent an ideal baseline point for the subsequent capture of digital signals using a composite monitoring platform (vide infra). This also represents a time when therapeutic options can be considered. Alongside the standard L-DOPA–based symptomatic therapies, there are myriad investigational disease-modifying drugs being evaluated, and the wider availability of well-described patient cohorts will be beneficial to increase diversity in the clinical trials and help uncover underlying principles accounting for the observed heterogeneity of the disease [41]. Coupled with the tenets of P4M, the likelihood of clinical success of these agents may be higher. Of note, the Critical Path for Parkinson’s Disease group (CPP) has outlined a drug development pathway that is intended to integrate traditional and digital measures in the holistic management of patients with PD. This consortium-based approach (CPP 3DT) is noteworthy as it embodies the tenets of P4M across the patient support structure, including carers and family members [42].

## The Role of Digital Devices

### Overview

Consumer-grade digital monitoring devices have begun to have a marked impact in the detection and management of health conditions [43]. As sensitivity, reliability, and interoperability of these data are refined, there is high expectation that they will factor into standard practice and serve as clinical outcome assessment tools relied upon to inform decisions made by health care providers (HCPs), health authorities, and payers. Consortia are already actively advocating for standardized approaches and parallel efforts are focusing on data security and regulations [44]. While multiple approaches can and will add value to the PD community at large, there seems to be logic in consideration of low-cost, ubiquitous devices that have a low burden of friction for the user. This latter consideration is especially relevant in PD, as deterioration in both motor skills and cognitive ability impair executive function over time. Two devices with high appeal are smartwatches and smartphones, with an ever-increasing array of capabilities added. For the purposes of illustration, herein, we focus on the smartphone (of which there are over 6 billion worldwide), but features are often later incorporated into smartwatches as sensors, computing power, battery life, and cost of goods fall. In the prodromal stages, there is a major opportunity to utilize the smartphone platform to detect early warning signals. The array of PD symptoms (Figure 1) span audible, motor, and movement, and combinations of signals could be analyzed and developed through personal-level artificial intelligence (AI)/machine learning processes to develop alerts [45]. Over time (months or years), these data captured longitudinally could form part of the individual’s health data through the smartphone or a third-party app and trigger discussions with their HCP during scheduled checkups. In the same way that early warning systems detect seismic activity and tremors underground, algorithms subsequently predict the degree of severity, duration, and interval of future events; thus, analogies in neurodegenerative disease and stroke are realistic. As an example, deviations in the REM sleep profile are an early warning sign for prodromal PD, and the opportunity for a smartphone to capture related acoustic changes passively is a very real possibility [46]. There are other components of the patients’ so-called *digital exhaust*, which might be useful in PD detection and management based on both passive and active device engagement [47].

### Smartphone Sensors Relating to PD

**Figure 4:**
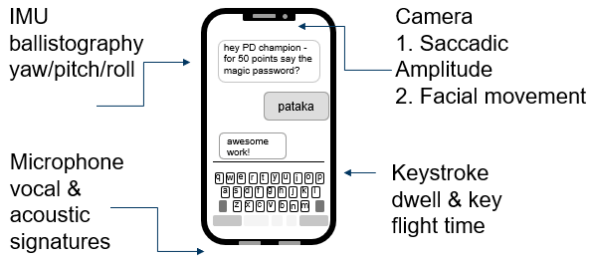
Parkinson disease–relevant digital measures from consumer-grade smartphones. IMU: inertial measurement unit.

Modern smartphones contain an array of sensors with relevance to the assessment of PD manifestations and their management (Figure 4). The inertial measurement unit (IMU) utilizes an accelerometer, magnetometer, or gyroscope to record movement and specifically for PD the angular rate and direction of movement. This functions across three axes (yaw, pitch, and roll) and if coupled to GPS sensors can track over distances, which could identify signals from short tremor bursts all the way through to balance impairment and ultimately falls. In another example, tremors associated with so-called “off” periods in patients with PD using symptomatic therapies might be detected, alerting them (and the HCP) to intervene [48]. The microphones on modern smartphones are now research-grade instruments and the devices are thus capable of detecting, interpreting, and recording both vocal and acoustic measures. This has been investigated heavily in PD with microfeatures able to discern between diseased states in cohorts [49]. Likewise, the virtual keyboards in smartphones have been used to identify hallmark signatures of PD through analysis of variables such as stroke pressure and interkey flight/dwell time in addition to specific aspects of what is typed, the frequency of such, and the error rates within [50]. A rapidly emerging tool is the use of the front camera for the analysis of features and idiosyncrasies in PD, which includes saccadic movement of the eye [51] and signature movements of the facial muscles, including the lips [52]. Each of these four classes of signals are powerful in their own right, and could form a time stamp of baseline to current to assess time-lapsed disease progression and the impact of therapeutics and P4 medicine interventions longitudinally, potentially over decades.

However, taken together, the potential power of *composite, orthogonal measures* is even higher [53]. Such methods offer many advantages over single-point analyses and offer the potential to help interpret individual data sets by providing contextual information. For example, a suddenly elevated heart rate prompting a rapid respiratory response and resulting acoustic signature may be explainable by GPS/IMU, which is trained by machine learning/AI to detect when the patient climbs long flights of stairs [53]. The use of orthogonal analytics is also supported by regulatory agencies such as the Food and Drug Administration (FDA) in the characterization of complex drug substances, requiring sponsors to demonstrate comparability through a totality of evidence (fingerprint) as opposed to isolated individual metrics [54]. Additionally, just as analytical methods improve over time, so will sensing technologies used to develop composite signals. These data can form a part of a powerful blueprint to empower the patient and their caregivers to take into discussions with their treating physician to recommend best actions. Digital interfaces and customized data repositories could form the basis of a form of “home hub” to guide disease management. It is of course expected that decisions are ultimately made by the patients’ HCPs who could be opted in to receive the personalized data sets. However, the process of patient participation in capturing these data is an attractive and essential component of the P4 medicine approach and could have a positive benefit in terms of engagement and positive outlook [55]. However, the trend in health care toward personal diagnostics goes far beyond that associated with smart devices. Specifically, there are emerging point-of-care and at-home diagnostics that could be of benefit to PD management and are thus worth highlighting.

### At-Home Analytical Methods

**Figure 5:**
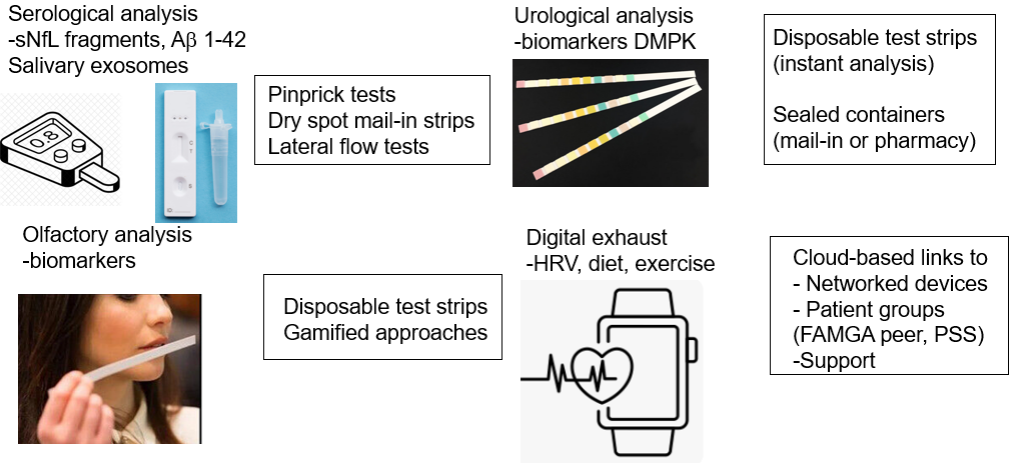
Potential Parkinson disease–relevant health measures attainable using at-home diagnostics. Aβ: amyloid β; DMPK: drug metabolism and pharmacokinetics; FAMGA: Facebook, Apple, Microsoft, Google, Amazon; HRV: heart rate variability; PSS: patient support services; sNfL: serum neurofilament light chain.

The growth of remote medical testing tools has become pronounced recently and accelerated by the COVID-19 pandemic. The utility of a simple-to-use disposable diagnostic device with colorimetric output was first demonstrated with at-home pregnancy test kits and later refined for devices for the rapid detection of antibodies such as in SARS-CoV-2 variants, which subsequently grew into a US $20 billion industry [56]. Part of the attractiveness is that the analytes (urine flow and saliva, respectively) are accessible noninvasively and the readouts are simple, being binary in nature. In terms of analysis of diagnostic, prognostic, and maintenance biomarkers for PD, there may be near-term potential for this approach. The first of these could be serology-based. For example, fragments of two of the signature proteins associated with PD are found in plasma in addition to CSF (neurofilament light chain and amyloid-β_1-42_) [57]. Although it may be unrealistic currently to effect point-of-care quantitation of these proteins (eg, through an antibody flow assay on a glucometer-style device), many of the barriers to pinprick blood sampling have been overcome and a hybrid approach may be feasible, with the patient mailing in or dropping off a device at a local pharmacy for subsequent quantitation. Of note, exosomal markers of amyloid proteins associated with AD and PD have been detected in saliva, which could ultimately lead to a COVID-19 type binary test (Figure 5) [58].

Another hallmark of early stage PD is the impact on olfactory sensing [59]. For example, patients may be encouraged to report on sensing ability if supplied with a series of test strips (similar to those used in a perfumery) periodically. Results could be captured by a gamified prompt on a smartphone app and the data fed in to the powerful composite analytics described above. In this manner, the voice analysis could be considered passive with respect to the main task of describing smell, which could have additional advantages in terms of unbiasing [60]. There is also evidence that olfactory analysis of the sebum of patients with PD is a powerful indicator of a diseased state, suggesting that self-captured sebum could be used for analysis and potentially help in classifying the disease subtype (Figure 2) [61]. The field of remote urinalysis has also developed rapidly in recent years. Originally, in the purview of dedicated analytical testing laboratories (eg, employer-based drug testing), many of these procedures have been adapted for the consumer market. One such example is the Test Card system, which offers a colorimetric readout for urinary metabolites that can be in the form of specifically tailored biomarkers [62]. The visual image is captured and digitized using a smartphone, thus potentially feeding directly into a digital health record. Such a system could be useful for analyzing drug metabolism and pharmacokinetics of metabolites of symptomatic- and disease-modifying therapies, newly discovered biomarkers, or explaining any contraindications encountered by polypharmacy connected with treatments for comorbidities. Other variants exist; for example, the FDA approved a mail test kit developed by dip.io for biomarker analysis [63].

**Figure 6:**
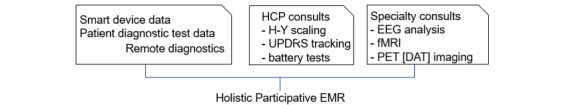
Integrating consumer-generated and health provider data sets. EEG: electroencephalogram; EMR: electronic medical record; fMRI: functional magnetic resonance imaging; H-Y: Hoehn and Yahr; PET [DAT]: positron emission tomography dopamine transporter scan; UPDRS: Unified Parkinson’s Disease Rating Scale.

Finally, one can also consider integration of signals from other devices the patient has access to. This could include data from smartwatches, fitness bands, smart rings, or even Internet of Medical Things devices deployed in the home (smart scales, smart toilets, voice assistants). Also important could be links through social media not accessed through the smartphone (eg, frequency of interacting in chat groups on a personal computer), as this behavior has been tied to gauge social well-being and detection of depressive episodes [64]. A possible approach to integrate these data sets as part of the patient’s electronic health record is depicted in Figure 6.

## Summary and Next Steps

The above approaches and opportunities appear to have the potential to refine how PD is diagnosed and treated and how patients are monitored and ultimately supported. There is an effective balance required for success that will demand careful deliberation, but a potential upside is that it could help codify improvements in quality of life for patients with PD across cognitive and motor dimensions. In the future, such data—which might be measurable over 1-, 6-, and 12-month time frames—could form the basis of informed decisions for HCPs and payers on treatment and prescribing actions, which, from recent experience with AD therapeutics, is a critical consideration to drive action [65]. Integrating patient-captured data into individual electronic health records would require adherence to predefined standards but could form a natural component of holistic care (Figure 6, left panel). The benefits of continual (patient) monitoring versus episodic (physician) monitoring would of course depend on the stage of disease (Figure 1) and the patient’s appetite for active engagement, but the potential upside could be considerable. Having statistically relevant composite measures could also provide a degree of granularity, which might serve to motivate certain patients. For example, a patient may feel disenfranchised learning that they have at best a maintained level or only slightly increased score on a broad assessment scale (eg, Unified Parkinson’s Disease Rating Scale, Hoehn and Yahr scale), but if a signal for real improvement in one or more digital subscales could be demonstrated, this might serve to motivate improved adherence and persistence levels [66]. The psychology of reward-driving behaviors is well understood in the consumer products and leisure industry and some of these learnings may help optimize engagement [67].

To establish the approaches described herein at scale would require close association with patient groups, advocates, HCPs, and platform technology developers. What is clear, however, is that the relentless development of sensitive and impactful analytic technologies will continue to offer new opportunities for the PD community. Already, the potential for AI approaches to personalization has been proposed [68]; the impact of new automated speech recognition technologies appears to hold great promise in detection and staging in PD [69,70] and the utility of chatbots such as GPT3 is already being pursued in neurodegenerative disease [71]. Interrogation of speech and acoustic signals as components of composite measures is also gaining traction, including with facial analysis [72], finger tapping [73], and nocturnal breathing events [74,75]. Efforts to integrate such measures alongside conventional assessments and interventions will help ensure their wider acceptance, and there is already progress correlating patient acoustic signatures with electroencephalogram (EEG) analyses [76] and the impact of symptomatic treatments on digital signal fidelity [77]. Continual technological advances, including in-ear EEG [78], muscle tremor detectors [79], and new approaches to speech interrogation [80], promise to push boundaries yet further and offer additional tools for patient assessment and symptom management.

In conclusion, the unifying theme we believe necessary to advance the opportunities and approaches described herein is the capture and storage of these health data digitally. This can ensure that appropriate decisions are made by patients and physicians longitudinally over the decades of the disease. Perhaps never before has such an opportunity presented itself for the use of consumer devices for long-term disease management. These are certainly exciting times and it is incumbent on us to ensure that opportunities are pursued logically and systematically so that patients with PD can derive personalized benefit in their lifetime.

## Abbreviations

AD: Alzheimer disease
AI: artificial intelligence
CSF: cerebrospinal fluid
CPP: Critical Path for Parkinson’s
EEG: electroencephalogram
FDA: Food and Drug Administration
HCP: health care provider
HD: Huntington disease
IMU: inertial measurement unit
LBD: Lewy body dementia
L-DOPA: levodopa
PD: Parkinson disease
PM: personalized medicine
PPMI: Parkinson's Progression Markers Initiative
P4: predictive, preventative, personalized, and participatory

## Data Availability

All data utilized in the preparation of this manuscript were obtained from publicly available sources listed in the references section.
